# Invasive pneumococcal disease in the Gulf region: a narrative review of incidence, burden, and vaccine strategies

**DOI:** 10.3389/fpubh.2025.1589366

**Published:** 2025-09-05

**Authors:** Eiman Mokaddas, Amina Khalfan Al-Jardani, Nermin Kamal Saeed, Sanjay Doiphode, Abiola Senok, Jean Joury, Mohamed Egaila

**Affiliations:** ^1^Microbiology Department, Faculty of Medicine, Kuwait University, Kuwait, Kuwait; ^2^Central Public Health Laboratories, Center for Disease Control and Prevention, Ministry of Health, Muscat, Oman; ^3^Medical Microbiology Section, Pathology Department, Salmaniya Medical Complex, Ministry of Health, Manama, Bahrain; ^4^Microbiology Department, Royal College of Surgeons in Ireland – Medical University of Bahrain, Busaiteen, Bahrain; ^5^Weill Cornell Medicine-Qatar, Hamad Medical Corporation, Doha, Qatar; ^6^College of Medicine, Mohammed Bin Rashid University of Medicine and Health Sciences, Dubai, United Arab Emirates; ^7^School of Dentistry, Cardiff University, Cardiff, United Kingdom; ^8^Pfizer Gulf FZ LLC, Dubai, United Arab Emirates

**Keywords:** invasive pneumococcal disease, *Streptococcus pneumoniae*, gulf region, epidemiology, serotype distribution, risk factors, vaccination strategies

## Abstract

Invasive pneumococcal disease (IPD) presents a significant public health challenge in the Gulf Cooperation Council (GCC) region, particularly affecting vulnerable populations such as young children and individuals with chronic conditions. This narrative review synthesizes recent data from Bahrain, Kuwait, Oman, Qatar, Saudi Arabia, and the United Arab Emirates (UAE) to assess the epidemiology of IPD, the burden of disease, serotype distribution, and risk factors. Across the GCC, there is a notable variability in serotype distribution, with non-Pneumococcal Conjugate Vaccine (PCV) 13 serotypes like 8, 15B, 22F, 33F, etc. becoming prominent. In Kuwait, PCV13 provided 61.5% serotype coverage, while in Oman, coverage is limited to 37.1%. Qatar reports higher coverage of 78.26% for children under two, however PCV13 strains such as 3 and 19A remain dominant. Antibiotic resistance is rising in several countries, especially in Kuwait and the UAE, where multidrug-resistant strains are becoming more common. The review also highlights the challenges posed by socioeconomic factors, including limited healthcare access, particularly among expatriates. While PCV13 has led to reductions in vaccine-covered serotypes, the emergence of non-PCV13 serotypes suggests the need for higher-valency vaccines like PCV20. Strengthening surveillance, expanding local research, and improving vaccination strategies tailored to the region’s unique demographic and healthcare landscape are essential for mitigating IPD burden.

## Introduction

Pneumonia remains a global health challenge, contributing to substantial morbidity and mortality, particularly among vulnerable populations (1). In 2021, pneumonia and other lower respiratory tract infections (LRTIs) were responsible for approximately 2.18 million deaths globally, with the highest mortality rates observed in children under five, older adults over 70 years, and individuals with comorbidities such as chronic lung disease, diabetes, or immunodeficiency (1–3). Among the various causative agents, *Streptococcus pneumoniae (S. pneumoniae)*, is a major etiological agent for both invasive pneumococcal diseases (IPD) and noninvasive illnesses, accounting for a large portion of pneumonia-related mortality (4, 5). IPD encompasses a spectrum of severe illnesses, including meningitis, bacteremia, septicemia, and bacteremic pneumonia, which present a high risk of fatality, particularly in younger and older age groups (4, 6, 7).

The pathogenesis of IPD typically begins with the prior colonization of the nasopharynx by *S. pneumoniae*, highlighting the importance of understanding both the epidemiology and serotype distribution of this pathogen (7–9). The polysaccharide capsule, which serves as the primary virulence factor, contributes to the diverse global landscape of pneumococcal serotypes. There are 94 identified serotypes, which exhibit significant variation based on factors such as age, ethnicity, and geography. However, a subset of 20 serotypes consistently accounts for the majority of IPD cases, accounting for more than 80% of cases across different age groups. Within this group, 13 serotypes are particularly responsible for 70–75% of invasive diseases in children (7, 10, 11). This overlap highlights the importance of focusing preventive measures on these high-burden serotypes, especially in vulnerable populations. Recognizing this, the World Health Organization (WHO) has recommended the inclusion of PCVs in national immunization programs, including pneumococcal conjugate vaccines (PCV) PCV-7, PCV-10, and PCV-13, offer protection against specific serotypes implicated in IPD (12–14) This concentration of disease burden within a limited number of serotypes underpins the development of PCV which target specific serotypes to reduce disease transmission and mortality (11, 15).

The effectiveness of vaccination strategies is challenged by the emergence of antimicrobial resistance (AMR) and non-vaccine serotypes (NVTs), which complicate both prevention and treatment efforts. This highlights the need for broader vaccine coverage and improved surveillance systems to better monitor resistance patterns (15–17). Surveillance data indicate growing resistance of *S. pneumoniae* to various antimicrobial agents, including penicillin, cephalosporins, macrolides, and quinolones. This upward trend in AMR emphasizes the importance of expanding vaccine coverage and enhancing surveillance systems (17–19). The advent of vaccines like PCV7, PCV10, and PCV13 has revolutionized the fight against pneumococcal disease. However, the emergence of AMR and NVTs has prompted the need for broader vaccine coverage, such as the newly approved PCV20 (15).

In light of these global challenges, the Gulf region has also made significant strides in addressing pneumococcal disease through the introduction of vaccines, although challenges remain. Recent studies in the United Arab Emirates (UAE) highlight the notable incidence of LRTIs, particularly among older adults, resulting in over 1,000 reported cases annually. To mitigate this issue, the Dubai Health Authority recommends a sequential vaccination strategy, beginning with PCV13 followed by the 23-valent pneumococcal polysaccharide vaccine (PPV23) for at-risk adults (20). Furthermore, the newly approved PCV20 shows promise in reducing pneumococcal disease and associated mortality (20). In Kuwait, the national vaccination program began with the PCV7 in 2007, transitioning to PCV13 in 2010 for children under 2 years. However, recent findings suggest that PCV13 has had limited impact on non-PCV7 serotypes, indicating need for refined vaccination strategies. Despite this, significant reductions in disease have been observed for PCV7 serotypes, emphasizing the ongoing need for vaccine evaluation in the region (21). Similarly, Qatar continues to recommend PPV23 alone for at-risk adults, while other Gulf countries, such as Kuwait, have adopted sequential vaccination strategies (PCV followed by PPV23) to address the diverse serotype distribution (21, 22). Oman began introducing PCVs with PCV7, followed by PCV10 and PCV13 in 2012, to expand serotype coverage. The transition to PCV13 was instigated by the emergence of multidrug-resistant (MDR) 19A serotypes. Additionally, adult high-risk groups in Oman are offered PPV23 to further mitigate the impact of pneumococcal disease (12).

Despite progress in vaccine deployment and surveillance efforts, gaps remain in understanding the burden of pneumococcal disease and AMR trends in the Gulf Region. The dynamic population demographics and mobility in the region further complicates disease surveillance and control. Therefore, there is a critical need for continued research and surveillance to inform evidence-based interventions and optimize preventive strategies against pneumococcal disease in the Arabian Gulf region (17, 23, 24).

This review aims to provide a comprehensive analysis of IPD within the Gulf Cooperation Council (GCC) countries of the Arabian Gulf region. By focusing on the epidemiology, burden, serotype distribution, and risk factors of IPD, this study highlights the prevalence and types of diagnosed cases across various age groups, as well as the distribution of the most common *S. pneumoniae* serotypes causing the disease. Additionally, it examines key risk factors among affected populations and evaluates the historical and current efficacy of vaccination programs in reducing the incidence of IPD, particularly within high-risk groups. The scope of this narrative review spans all GCC nations, offering a detailed comparative analysis to inform future public health strategies.

**Search Strategy**: A comprehensive literature search was conducted in PubMed to identify studies on IPD in the GCC region (Bahrain, Oman, Kuwait, Saudi Arabia, Qatar, UAE,). We used keywords such as “pneumococcal infection,” “*Streptococcus pneumoniae*,” “invasive pneumococcal disease,” “bacteremia,” “meningitis,” and “pneumonia,” along with geographic terms related to the GCC. Studies were included if they focused on the epidemiology, serotype distribution, vaccine coverage, or burden of IPD. Primary research and review articles were considered; studies outside the Gulf region or focused solely on non-IPD were excluded. A narrative synthesis approach was employed to summarize key findings due to the heterogeneity of the data.

## Epidemiology of pneumococcal disease in the Arabian gulf region

**Bahrain**: Epidemiological data from Bahrain indicate a significant burden of IPD, particularly among young children, particularly in young children, with children under 2 years accounting for the majority of cases. A demographic analysis study showed a male-to-female ratio of 1.4:1, with 44% of patients being children aged five and under, and 34% in the 16–64 age group (25). In a study which examined epidemiology over a five-year period before the introduction of the PCV, 371 cases of IPD were reported in children under 5 years. The majority of these cases were male (mean age 1.25 years), and a significant portion (73.9%) occurred in children aged 0–1 year. The annual incidence ranged from 41.7 to 207.6 per 100,000 children under 5 years, with an increase observed from 1999 to 2003. Children under 2 years accounted for 83.8% of the cases, with a corresponding rise in meningitis, septicemia, and pneumonia (26). A longitudinal study by Saeed N, et al. revealed a decline in pneumococcal meningitis cases, dropping from 1 to 0.1 per 100,000 population between 1990 and 2013 possibly due to the introduction of the PCV and growing vaccination uptake (27). PCV was introduced in Bahrain in 2002 for high-risk groups and was extended to the entire population in 2008 (28).

**Kuwait**: A hospital-based study between 2010 and 2014 found that out of 196 meningitis cases, 29% were bacterial, equating to 57 cases. Among these, 23 (40.4%) were due to *S. pneumoniae*. Notably, 48% patients with pneumococcal meningitis had not completed the full PCV series (29), highlighting gaps in vaccine coverage. Although the introduction of PCV7 in 2006 and PCV13 in 2010 has impacted disease incidence, incomplete vaccination continues to contribute to the disease burden. Additionally, the bacterial meningitis rate of 13 per 100,000 individuals was observed from 1981 to 1987 had 21% of cases attributed to *S. pneumoniae*. The highest annual frequency of invasive pneumococcal isolates reported after the introduction of PCV7 (28.50 isolates/year), compared to 20.67 isolates/year before PCV introduction and 16.04 isolates/year post-PCV13 (30).

**Oman**: In Oman, PCV7 was the first PCV introduced in 2008 (12, 14). For children under 2 years, the incidence was 26.1 per 100,000, while for those aged 2 to 5 years, it was 12.3 per 100,000, and for children under 5 years, it was 18.6 per 100,000. In total, 59% of IPD cases occurred in children under 2 years, with clinical manifestations of meningitis (34%) and septicemia or bacteremia (66%). Antibiotic resistance to at least one antibiotic was observed in 54% of *S. pneumoniae* isolates. Additionally, the incidence among children in Oman ranged from 7.7 to 17.4 per 100,000 in 2006 (28, 31). Despite the introduction of PCV, IPD remains a concern, particularly with emerging serotype changes and AMR. Another study spanning 9 years reported 581 suspected or confirmed meningitis cases with a male preponderance (61%) and majority (71%) being children under the age of 13 years. Of these, 55% were children below 2 years old, and 54 were neonates. Bacterial meningitis constituted 66% of cases, with a decline observed in pneumococcal meningitis reports in later years, possibly reflecting the impact of vaccination programs. While the decline in pneumococcal meningitis in Oman over recent years could reflect the impact of ongoing vaccination efforts, further studies are necessary to confirm the role of vaccination in this reduction (32).

**Saudi Arabia**: PCV7 was introduced into Saudi Arabia’s National Immunization Program in 2009 and was later replaced by PCV13 in 2010. A catch-up vaccination campaign for children aged 2–5 years in 2015 led to a 98.7% PCV uptake, significantly reducing the incidence of IPD (30). Between 2007 and 2009, a study examined 1,030 episodes, of which 631 episodes were recorded as suspected IPD, and 623 episodes involving 622 children were analyzed. Among these, 7.2% were in infants under 1 month, 46.5% in those aged 1 month to 1 year, and 46.2% in children aged 1 to 5 years. The study confirmed *S. pneumoniae* in 7.5% of suspected cases, with incidence rates of IPD in children under five ranging from 2.5 to 9.6 per 100,000, with a notably higher rate of 21.6 per 100,000 in Al-Baha City. The low vaccination rate (0.2%) among children with suspected IPD highlights the importance of expanding PCV coverage (33). These findings suggest that increasing vaccination uptake could reduce the burden of pneumococcal disease, particularly among vulnerable populations.

**United Arab Emirates (UAE)**: PCV-13 was introduced in the UAE in 2011 (17). Over a 5-year period, 739 admissions for pneumococcal disease were recorded, with 5.4% classified as invasive disease (approximately 40 cases of IPD). The calculated incidence rate of 13.58 per 100,000 children under 5 years old annually was derived from data collected from Mafraq hospital and SKMC in Abu Dhabi, which together represent 80% of the pediatric population under 5 years in the emirate. In 2005, the pediatric population of Abu Dhabi was estimated at 92,050 children, and the denominator used for calculating the incidence was 73,640 children (based on hospital catchment areas). Most cases (57.5%) occurred in children under 2 years. Furthermore, the higher mortality rate and longer hospitalization durations among IPD cases compared to non-IPD cases highlight the severity of the disease and the need for effective prevention strategies, including vaccination (18). Another study of 361 pneumonia patients in the UAE, primarily from Abu Dhabi, revealed significant clinical and demographic insights. The cohort, predominantly UAE nationals (68%), showed an increase in annual community-acquired pneumoniae (CAP) admissions from 250 to 710 per 100,000 between 1997 and 2002, with CAP peaking in March and April. Common co-morbidities included diabetes (21%), malignancy (20%), and COPD (19%). The overall pneumonia mortality rate was 13%, higher for hospital-acquired pneumonia (HAP; 24%) than CAP (10%). This study highlights the rising hospitalization rates for CAP in the UAE, particularly in Abu Dhabi, influenced by seasonal variations and possibly by the movement of expatriate populations (34). Another study by Senok et al. (17) based on 12-year national surveillance data from 2010 to 2021 for AMR in pediatric and adult patients provided insights into the demographic distribution and clinical outcomes of pneumococcal disease in both pediatric and adult populations in the UAE. Male predominance was observed among patients, with a higher occurrence of hospitalization and mortality rate among IPD patients compared to non-IPD patients. Notably, there was a shift in the demographic trend over time, with a decline in the percentage of Emirati nationals affected by both IPD and non-IPD. AMR trends indicated a decrease in penicillin G resistance but an increase in resistance to cefuroxime, levofloxacin, and moxifloxacin, with a significant rise in MDR isolates from 16.4% in 2013 to 42.2% in 2021 (17). These findings underscore the importance of continuous surveillance and targeted interventions to address the evolving epidemiology of pneumococcal disease.

**Mass Gatherings**: The prevalence of pneumococcal infections during Hajj varies widely, with pneumonia cases caused by *S. pneumoniae* ranging from 1 to 54% (35). This variability highlights the critical need for targeted vaccination and surveillance strategies, particularly since CAP is predominantly due to *S. pneumoniae* (35, 36). Research on Hajj pilgrims have shown a significant rise in MDR strains of *S. pneumoniae* post-pilgrimage, suggesting that Hajj may contribute to the spread of AMR. MDR strains exhibit a higher phenotypic diversity in post-Hajj samples, indicating the acquisition of new resistant strains during the pilgrimage (37). These findings are indicative of the importance of targeted vaccination and surveillance strategies during mass gatherings such as Hajj. Pneumococcal vaccination has been recommended for pilgrims over 50 years old, with the Saudi Ministry of Health emphasizing vaccine uptake among high-risk populations (38). However, vaccination rates remain suboptimal, with discrepancies in vaccine coverage observed among pilgrims from different regions (39).

The epidemiology of pneumococcal disease across the Gulf region shows varying trends, influenced by vaccination coverage, disease incidence, and regional public health strategies. (Table 1). It remains a major health concern, with the highest disease burden consistently observed in children under 2 years old. In Bahrain, the incidence of IPD has decreased significantly post-PCV introduction, particularly in children under 2 years, reflecting the effectiveness of vaccination. Similarly, Oman has also witnessed a decline in pneumococcal meningitis cases, and available data suggests that this reduction may be due to ongoing vaccination efforts and further studies are needed to confirm this. The UAE also reports high incidence rates of IPD in young children, underscoring the vulnerability of this age group. In Saudi Arabia, IPD rates remain concerning, with a notably low vaccination rate among children, pointing to the need for expanded vaccine coverage. Similarly, in Kuwait, gaps in vaccination coverage are evident, with a high proportion of pneumococcal meningitis cases occurring in unvaccinated individuals, further emphasizing the need for improved vaccine uptake. Mass gatherings, such as Hajj, exacerbate the spread of pneumococcal infections, particularly MDR strains. Despite ongoing vaccination efforts, inconsistent vaccine coverage and limited surveillance continue to pose significant challenges. Overall, although vaccination programs have made notable progress in some countries, disparities in coverage, gaps in surveillance, and the growing threat of AMR contribute to varying patterns of IPD epidemiology reported across the Gulf states.

**Table 1:** Epidemiology of pneumococcal disease in the Arabian Gulf region.

Country	Key epidemiological data	Key findings
Bahrain (14, 25, 26, 27, 28)	Male-to-female ratio: 1.4:1 44% of IPD cases in children <5 years IPD incidence highest in children <2 years PCV introduced in 2002 (risk groups) and 2008 (universal)	Majority of IPD cases in children under 5 years (73.9%) Significant increases in meningitis, septicemia, and pneumonia Decrease in pneumococcal meningitis post-PCV introduction
Kuwait (21, 29, 30, 40, 41, 42, 45)	29% of meningitis cases bacterial (*S. pneumoniae*: 40.4%) PCV7 introduced in 2006 and PCV13 in 2010	Gaps in vaccine coverage among high-risk groups Decrease in invasive pneumococcal isolates after PCV introduction
Oman (12, 14, 28, 31)	IPD incidence <2 years (26.1 per 100,000) IPD: Meningitis (34%) and bacteremia (66%) 54% of isolates resistant PCV7 introduced in 2008	Significant reduction in pneumococcal meningitis in recent years, possibly due to vaccination High resistance rates in *S. pneumoniae*
Saudi Arabia (30, 33)	7.2% of suspected IPD cases confirmed (*S. pneumoniae*: 7.5%) Higher incidence in Al-Baha City (21.6/100,000) PCV7 introduced in 2009 and PCV13 in 2010	Low vaccination rate (0.2%) in children with suspected IPD Increased vaccination uptake may reduce disease burden
United Arab Emirates (17, 18, 34)	57.5% of cases in children <2 years Mortality higher for IPD cases MDR strains (16.4% in 2013 to 42.2% in 2021) PCV13 introduced in 2011	Rising hospital admissions for CAP, particularly in Abu Dhabi Increase in multidrug resistance, necessitating enhanced surveillance and interventions
Mass Gatherings (Hajj) (35, 39)	Prevalence of *S. pneumoniae* pneumonia: 1 to 54% ICU admissions mainly due to non-*S. pneumoniae* infections	High prevalence of pneumococcal infections during Hajj Significant rise in MDR *S. pneumoniae* strains post-pilgrimage

CAP, community-acquired pneumonia; IPD, invasive pneumococcal disease; MDR, multi-drug resistant; PCV, pneumococcal vaccine.

## Burden of pneumococcal disease

**Bahrain**: Pneumococcal infections in Bahrain pose a considerable burden, particularly among young, the older adults, and those with chronic illnesses. These patients often require intensive medical care and hospitalization. The true incidence and burden of IPD are likely higher due to underreporting. From 1999 to 2003 a notable increase in incidence was observed, especially among children under 2 years old, who also face high mortality rates. Implementing active national surveillance is essential to evaluate vaccination program outcomes and control disease spread (26).

**Kuwait**: Kuwait faces challenges in treating pneumococcal infections due to AMR rates exceeding 40% for several commonly used antibiotics. Despite susceptibility to certain antibiotics like levofloxacin, vancomycin, and linezolid, the overall resistance pattern remains a concern. Efforts to combat AMR include hospital-based antibiograms and annual campaigns for proper antibiotic use. However, there is a need for national guidelines and policies to effectively address the problem (40, 41). A retrospective study of childhood meningitis cases, following the introduction of the pneumococcal conjugate vaccine (PCV), found that these cases required an average hospital stay of 13 days. Among the 196 patients, 33 required intensive care, 21 needed artificial ventilation, and 2.5% died. Additionally, 3% of survivors suffered from long-term complications like seizures, hydrocephalus, or motor palsy (29). This represents a significant burden on healthcare resources, indicating the considerable medical attention and prolonged recovery time required for individuals affected by IPD. In a study by Mokaddas et al. (42) the antibiotic susceptibility profiles of *S. pneumoniae* isolates were varied, with some showing resistance to penicillin, erythromycin, and azithromycin. MDR was observed in isolates from cerebrospinal fluid (CSF) and blood samples. Analysis of genetic relationships via multilocus sequence typing revealed unrelated singletons and two clusters, ST1390 and ST17771, distributed across Kuwaiti hospitals. Interestingly, Kuwaiti isolates clustered with those from neighboring countries, suggesting regional genetic similarities. This information contributes to understanding antibiotic resistance and genetic diversity in *S. pneumoniae* across the Gulf region, informing targeted interventions (42).

**Oman**: The incidence of pneumococcal disease in Oman among children ranged from 7.7 to 17.4 per 100,000 population in 2006 (28, 31). Patients with pneumococcal meningitis require longer hospital stays and more intensive care compared to those with septicemia or bacteremia. Antibiotic treatment typically lasts over 10 days, with co-amoxiclav, ceftriaxone, and cefuroxime sodium being commonly used (12, 31). Despite the introduction of PCV in 2008, IPD remains a concern due to shifts in serotype prevalence and the rise of AMR, signaling ongoing challenges in managing pneumococcal disease (12).

**UAE**: In the UAE, pneumococcal disease imposes a significant burden, with high mortality rates, particularly among children aged <5 years. Although the introduction of PCV has contributed to a reduction in morbidity and mortality, AMR continues to be a growing concern. Being highly cosmopolitan, the UAE faces challenges in controlling the spread of AMR pathogens due to dynamic population movements. Despite the need for heightened surveillance, there is a paucity of published literature on the burden of pneumococcal infections in the country, with only a few studies predating the introduction of PCV-7. Recent data indicate a sustained increase in pneumococcal isolates, though a sharp decline was observed in 2020 likely due to COVID-19-related interventions. The emergence of MDR strains emphasizes the importance of continued surveillance and antibiotic stewardship programs to address this growing threat (17). An economic analysis by Zayed et al. (20) demonstrated the potential benefits of vaccination strategies, particularly among expatriates in Dubai. However, challenges in pathogen isolation from blood cultures indicate the need for improved diagnostic techniques to better assess the burden of pneumococcal disease (20).

**Mass Gatherings**: AMR data from Hajj indicate significant challenges in controlling infections with an increase in MDR strains post-pilgrimage, contributing to higher treatment complexities and prolonged illness (37). These resistant strains complicate the management of pneumococcal disease, leading to increased healthcare costs, longer hospital stays, and higher morbidity rates. Pilgrims from regions with low pneumococcal vaccination coverage may be especially vulnerable to AMR strains due to inconsistent vaccine implementation and suboptimal antibiotic stewardship (38). The variability in AMR resistance rates across regions highlights the need for tailored surveillance strategies to more accurately assess the pneumococcal disease burden (43). Suboptimal vaccination uptake in certain regions, combined with gaps in antibiotic stewardship, contribute to the increased risk of AMR strains during the pilgrimage (38, 44). This shows the urgent need for comprehensive vaccination campaigns and strengthened surveillance to mitigate the growing burden of pneumococcal disease and its resistant strains in the Gulf Region.

The burden of pneumococcal disease in the Arabian Gulf region varies across countries, with differences in disease incidence, healthcare impact, and AMR. In Bahrain, the pneumococcal disease burden is high with notable increases in incidence observed prior to the introduction of PCV. However, the healthcare system continues to face challenges in managing these cases, and improved surveillance is needed to evaluate the outcomes of vaccination programs. In Kuwait, AMR is a significant challenge, with resistance rates exceeding 40% for several commonly used antibiotics, highlighting the ongoing medical burden. In Oman, although the incidence remains relatively low, patients, especially those with meningitis, require intensive care and prolonged hospitalization, which places a strain on healthcare resources. The UAE has seen a reduction in disease incidence following the introduction of PCV, but the rise of MDR strains poses a growing challenge, compounded by the country’s diverse expatriate population. The Hajj pilgrimage adds complexity, with mass gatherings contributing to the spread of AMR strains, increasing the risk of pneumococcal disease transmission and complicating disease management. These findings emphasize the urgent need for enhanced vaccination strategies, improved surveillance, and effective antibiotic stewardship programs to reduce the burden of pneumococcal disease and combat AMR in the Gulf region.

## Serotype distribution and vaccine coverage

**Bahrain**: The predominant serotypes of IPD identified in Bahrain were 19, 6, 23, 3, and 14, with significant penicillin resistance observed, particularly in children under five (34%) and the older adults (29%). These data were derived from hospital-based surveillance, which evaluated 100 *S. pneumoniae* isolates collected from inpatients and outpatients at Salmaniya Medical Complex and Bahrain Defense Force Hospital. The isolates were taken from various infection types, such as pneumonia, otitis media, sinusitis, conjunctivitis, meningitis, and septicemia. Isolates from blood or cerebrospinal fluid (CSF) were classified as invasive, while respiratory and other fluid samples were categorized as noninvasive. PCV13 covers all serotypes associated with invasive infections in children under two, offering an overall coverage rate of 66.7%, while in children aged two to five, the coverage rate was 69%. However, 33.3% of serotypes remain uncovered by PCV13. In contrast, PPV23 provided a broader range of serotypes, covering 91.4% of all isolates, with 100% coverage for invasive and erythromycin-resistant strains and 95.2% for penicillin-resistant isolates in individuals over 2 years old. These findings suggest that PPV23 could enhance vaccination strategies, especially for older populations and resistant strains in Bahrain (25).

**Kuwait**: In Kuwait, the introduction of PCV7 and PCV13 into the national immunization program has led to a notable reduction in vaccine-covered serotypes. In a study, strains were collected from sterile site specimens, including blood and CSF, from both children and adults at general hospitals, tertiary-care hospitals, and polyclinics across Kuwait from 2003 to 2016. The isolates were sent to the Pneumococcal Reference Laboratory at the Faculty of Medicine, Kuwait University, for serotyping using the Quellung reaction. A marked decline in the prevalence of serotypes 9 V, 14, 19F, and 23F was observed in the post-vaccination period, particularly in older age groups. However, the reduction in non-PCV serotypes causing Pneumococcal Necrotizing Pneumonia (PNP) serotypes was not statistically significant, and the prevalence of NVTs, such as 8, 12F, and 15B increased after the introduction of PCV13. The limited effect of PCV13 on non-PCV serotypes, along with the increase in NVTs, suggests that the additional six serotypes in PCV13 may provide limited protection against these emerging serotypes in Kuwait. Higher-valency vaccines, including PCV20, could potentially address the rise in emerging serotypes and offer broader coverage. In addition, following PCV7 introduction, the frequency of PCV7 serotypes in children under 2 years increased slightly (to 1.75 isolates/year), while after PCV13 introduction, a decrease was observed in PCV7 and PCV13-only serotypes. However, there was a notable rise in non-PCV13 serotypes after both PCV7 and PCV13 introductions, particularly after PCV13 (21, 30). In another study, PCV13 achieved 61.5% serotype coverage in children under 2 years of age and 70.8% in adults aged 51–60 (45).

**Oman**: Serotypes 19F, 6B, and 23F were historically dominant contributors to pneumococcal disease in Oman. Despite the inclusion of these serotypes in PCV13, penicillin susceptibility remains limited, with only 44% of strains demonstrating sensitivity, raising concerns about antibiotic resistance. Furthermore, mortality rates were notably higher among individuals infected with *S. pneumoniae*, particularly those infected with serotypes 19F and 1, which are commonly associated with fatal outcomes (46). Recent surveillance data reveals a shifting serotype landscape in Oman, with serotypes/serogroups 12 (8.3%), 15 (8.3%), 19F (7.6%), 3 (6.1%), and 19A (6.1%) emerging as common serotypes not covered by PCV13. This surveillance data was primarily collected from hospital-based studies, focusing on IPD cases, including both pediatric and adult patients. The isolates were collected across multiple health facilities, including regional and tertiary hospitals, providing a representative sample of the circulating strains in Oman. Although PCV13 offers protection against some of the most prevalent serotypes, such as 19F, 3, and 19A, it covers 37.1% of the circulating serotypes in Oman, indicating a potential need for broader coverage. This evolving serotype distribution underscores the need for ongoing surveillance and that broader vaccine formulations may be needed to enhance protection (12).

**Qatar**: In Qatar, prevalent serotypes associated with IPD were 3, 14, 1, 19A, 9 V, 23F, and 19F, with varying distribution by age group. The PCV-13 vaccine offers a coverage rate of 78.26% for children under 2 years and 66.67% for those aged ≥ 65 years. The strains were isolated from patients attending Hamad Hospital and emergency clinics at Hamad Medical Corporation between January 2005 and March 2009. This study included invasive strains from both pediatric and adult patients diagnosed with IPD, defined as the isolation of *S. pneumoniae* from normally sterile body sites, such as blood and CSF. The isolates were identified through conventional methods, including optochin sensitivity and bile solubility, with antimicrobial susceptibility testing done for key antibiotics. The findings reflect circulating strains within hospital settings, which provide a snapshot of the serotype distribution in patients with more severe disease (7). Additionally, Tsui et al. emphasized that while the introduction of PCV-7 and PCV-13 has reduced vaccine-serotype related IPD, NVTs have been on the rise. They speculated that capsular switching and serotype replacement might contribute to this shift, reinforcing the importance of ongoing monitoring to evaluate the evolution of circulating serotypes and guide vaccination strategies (47).

**Saudi Arabia**: In a multicenter, prospective surveillance study by Almazrou et al., conducted across 12 hospitals in seven health areas, specimens were collected from children under 5 years suspected of IPD, including both hospitalized patients and outpatients (blood, CSF). Although this hospital-based study provided important insights into serotype distribution and vaccine coverage, it did not include data from community or school-based populations, limiting the broader applicability of the findings. The pneumococcal serotypes in Saudi Arabia were categorized into those covered by PCV7, PCV13, and those outside both formulations. PCV13 provided protection against serotypes 1, 3, 4, 5, 6A, 6B, 7F, 9 V, 14, 18C, 19A, 19F, and 23F while the non-PCV13 serotypes observed in the study included 8, 10A, 11A, 12F, 15B, 22F, and 33F. A decline in PCV13-covered serotypes was observed post-introduction of the vaccine; however, there was a simultaneous increase in non-PCV13 serotypes, suggesting a shift in the epidemiology of pneumococcal infections. The study’s scope was limited to a representative hospital sample, and the effect on serotype prevalence in other regions or age groups was not fully captured, making it difficult to generalize the findings. Additionally, trends in AMR and non-invasive disease cases were not comprehensively assessed. These limitations underscore the need for expanded national surveillance and consideration of broader vaccine coverage (33).

**UAE**: Due to lack of serotype-specific data, accurate estimates for pneumococcal serotype coverage remain limited. Zayed et al. (20) modeled the clinical and economic impact of different vaccination strategies in Dubai, specifically among expatriate populations aged 19–99 with varying risk profiles. This model relied on hospital-based data and did not account for strains circulating in school children or broader community settings. As such, the serotype distribution and epidemiology in these broader populations remain unclear. The estimates for pneumococcal serotype coverage in the study were based on surveillance data from Oman (2014–2016) and Canada (2013), highlighting the need for region-specific data to better inform vaccination strategies. The study suggested that using PCV20 instead of current PCV13/PPSV23 regimen may reduce IPD and pneumonia cases, saving up to $4.8 million in healthcare costs. The broader serotype coverage and simplified dosing schedule of PCV20 could contribute to improved vaccine uptake and offer more comprehensive protection to high-risk populations in UAE. However, basing policy decisions on proxy data presents limitations, especially in a country with unique demographic and healthcare dynamics. More comprehensive community-based and school-based surveillance studies are needed in the UAE to better understand serotype prevalence across diverse population groups and to develop more targeted vaccination strategies (20).

**Mass Gatherings**: During Hajj, specific disease-causing serotypes among pilgrims remain poorly documented, although carriage studies suggest common serotypes such as 3, 6A, and 19F, though a comprehensive understanding of the circulating strains remains elusive (48). The serotypes causing disease during Hajj have not been well defined, and isolation of *S. pneumoniae* through culture is not common, especially for CAP. A study screened 3,202 pilgrims from 18 countries found that PCV10 covered just 19%, PCV13 covered 38%, and PPV23 provided 55% coverage of the serotypes identified. Serotype data from Saudi Arabia show that serotypes 3, 6A, and 19F dominate among both pilgrims and the general population. These serotypes are also among the most common in Saudi Arabia and are covered by both PCV13 and PPV23 vaccines. However, serotype replacement due to the introduction of vaccines, like PCV13, has shifted the serotype landscape, and NVTs are becoming more prevalent (43). Vaccine coverage among Hajj populations, particularly older adult pilgrims who are at greater risk of severe disease, remains suboptimal despite recommendations, especially in countries with lower coverage (38, 49). This emphasizes the need for improved vaccination strategies targeting the most prevalent and AMR serotypes, as the variability in serotype distribution during Hajj, influenced by the diverse origins of pilgrims from countries with varying pneumococcal epidemiology, highlights the importance of adapting regional vaccination strategies to local epidemiology (39). Given the potential for serotype shifts and AMR, more targeted vaccination interventions are necessary to reduce the burden of pneumococcal disease, particularly among vulnerable populations.

The serotype distribution and vaccine coverage for pneumococcal disease in the Gulf region exhibit notable differences across countries, reflecting variations in vaccination strategies, emerging serotypes, and antibiotic resistance patterns. In Bahrain, PCV13 provides moderate coverage (66.7% for children under two), but the broader PPV23 offers more extensive protection, covering 91.4% of isolates, indicating the need for enhanced vaccine strategies, particularly for older populations and resistant strains. In contrast, in Kuwait, the introduction of PCVs has reduced the prevalence of vaccine-covered serotypes, but non-PCV serotypes, such as 8, 12F, and 15B, have emerged, suggesting a limitation in the broader protection offered by PCV13. Similarly, Oman also faces a shifting serotype landscape, with PCV13 covering only 37.1% of circulating serotypes, signaling the need for broader vaccine options due to rising NVTs like 19A and 3. Although Qatar reports a higher coverage rate for PCV13 in children under two (78.26%) but similar to Kuwait and Oman, increase in NVTs has been reported, pointing to the evolving nature of pneumococcal epidemiology. This trend has also been shown in Saudi Arabia where the surveillance data indicates a shift toward non-PCV13 serotypes post-vaccine introduction, further emphasizing the need for expanded vaccine coverage beyond the current formulations. In the UAE, modeling studies suggest that transitioning to PCV20 could offer more comprehensive protection, but a lack of serotype-specific data underscores the need for further research, especially in community settings. Finally, mass gatherings like Hajj show a complex serotype distribution, with common strains such as 3, 6A, and 19F being prevalent, but gaps in vaccine coverage, particularly among older adult pilgrims, highlight the need for improved vaccination strategies targeting high-risk populations and addressing the rising threat of AMR. Thus, across the GCC countries, the shifting serotype patterns and varying vaccine coverages indicate the need for continuous surveillance and the potential benefits of higher-valency vaccines to address emerging pneumococcal strains (Table 2). In addition to invasive strains, non-invasive serotypes such as 6C, 11A, 21, 17F, and 23A are also prevalent across the region. These serotypes are commonly associated with colonization or less severe respiratory tract infections (50–54) (Supplementary Table 1).

**Table 2:** Serotype prevalence and vaccine coverage in GCC countries.

Country	Predominant serotypes	Vaccine coverage (PCV13/PPV23)	Emerging non-PCV serotypes
Bahrain (25)	19, 6, 23, 3, 14	66.7% (PCV13)	Data not available
Kuwait (21, 30, 45)	9 V, 14, 19F, 23F	61.5% (PCV13, children <2)	Increase in 8, 12F, 15B
Oman (12, 46)	19F, 6B, 23F, 12, 15, 19A	37.1% (IPD cases covered by PCV13)	Increase in 12, 15, 19A
Qatar (7, 47)	3, 14, 1, 19A, 9 V, 23F, 19F	78.26% (PCV13)	Increase in nonvaccine serotypes
Saudi Arabia (33)	1, 3, 4, 5, 6A, 6B, 7F, 9 V, 14, 19F, 23F	7.5% (IPD cases covered by PCV13)	Rise in non-PCV13 serotypes
United Arab Emirates (17, 20)	Serotype data not available	Data not available	Increase in multidrug-resistant strains

## Risk factors for IPD

Identifying risk factors for IPD within the Gulf region is essential for targeted prevention and management strategies tailored to the local context. Children under the age of 2 years are particularly vulnerable to IPD due to their immature immune systems (31, 34). Furthermore, chronic medical conditions such as diabetes, human immunodeficiency virus, chronic respiratory diseases, and increased exposure during mass gatherings such as the Hajj pilgrimage significantly increase the risk of IPD among adults in the Gulf region (26, 55). Environmental factors, including overcrowded living conditions prevalent in some Gulf countries, may contribute to the transmission of pneumococcal bacteria and subsequently increase the risk of IPD (32, 40, 41). Moreover, socioeconomic factors such as limited access to healthcare services and lower vaccination coverage rates among certain populations in the Gulf region may increase the risk of IPD. Additionally, lifestyle factors such as smoking and excessive alcohol consumption may weaken the immune system and predispose individuals to infections, including IPD (26). Studies highlight that CAP, particularly pneumococcal pneumonia, poses a significant economic burden, especially in hospitalized patients, and affects patients’ quality of life. Targeted vaccination for individuals with COPD, asthma, heart disease, diabetes mellitus, and those who smoke should be prioritized, regardless of the time of year (55). Moreover, regional studies underscore the impact of demographic factors on IPD risk, with a notable emphasis on the high prevalence of pneumococcal disease among expatriates in Gulf countries. The influx of expatriates, often from diverse backgrounds with varying healthcare access, adds complexity to IPD epidemiology and highlights the need for targeted interventions tailored to this demographic group (17, 20). Understanding the interplay of demographic dynamics, socioeconomic status, and healthcare access is paramount for devising comprehensive strategies to mitigate IPD risk across the Gulf region.

## Current research gaps

Despite advancements in pneumococcal vaccination and surveillance across the Arabian Gulf region, critical research and policy gaps remain:

*Inadequate Country-Specific Surveillance*: Most Gulf countries lack updated, comprehensive national data on IPD incidence and serotype distribution, particularly in adults and high-risk groups. For instance, UAE serotype data have been inferred from neighboring countries, which limits the precision of vaccine policy recommendations (20).

*AMR*: Although AMR is increasing, surveillance programs are fragmented. In Kuwait and the UAE, multidrug-resistant strains are rising, but ongoing molecular characterization and whole-genome sequencing are limited (17, 41).

*Underserved Populations*: The unique demographic structure including expatriate workers with diverse backgrounds and limited healthcare access requires tailored vaccination and prevention strategies. Research specifically targeting these populations is scarce (34).

*Vaccine Policy Evaluation*: There is a lack of long-term, region-specific studies assessing the effectiveness, cost–benefit, and serotype-specific impact of newer vaccines like PCV20. Preliminary economic data from Dubai suggest benefits, but broader studies are warranted (20).

*Mass Gathering Health Preparedness*: The impact of large-scale events like Hajj on pneumococcal transmission is poorly characterized. There’s minimal data on serotype dynamics during these gatherings, limiting targeted intervention planning (35, 49).

## Challenges and barriers

The implementation of pneumococcal vaccination in the Gulf region faces several barriers. These include low health awareness, especially about the burden in adults, and poor knowledge of vaccine benefits among both healthcare professionals and the public (24). Additionally, cost is a significant barrier, particularly for expatriate populations who pay out-of-pocket for vaccines. Inadequate physician recommendations and missed opportunities to administer vaccines during healthcare visits further contribute to low vaccination rates (56). Moreover, inconsistencies in healthcare policies and vaccine availability across countries create unequal access, especially for high-risk groups (24). Addressing these barriers is essential to improve vaccination coverage and reduce pneumococcal disease burden.

## Key messages

This narrative review synthesizes the current epidemiological landscape, serotype distribution, risk factors, and vaccination coverage related to IPD in the Gulf region. The findings consistently highlight a significant burden of IPD, particularly among vulnerable groups such as young children, the older adults, and individuals with chronic health conditions (7, 18, 31, 46). Although the introduction of PCVs, particularly PCV13, has contributed to reductions in disease caused by vaccine-covered serotypes, several emerging challenges persist (17). A key concern is the rise in NVTs, including 8, 12F, and 15B, which now account for an increasing proportion of IPD cases in countries such as Kuwait (21, 30). In Oman, for instance, PCV13 only covers 37.1% of currently circulating serotypes, showing a substantial gap in protection (12). The trend toward serotype replacement calls for updated immunization strategies, including broader-valency vaccines like PCV20, which may address these emerging gaps.

The Gulf region’s vaccination coverage is still variable. While Qatar reports relatively high serotype coverage for children under two (78.26%) (7), data from the UAE are limited, often relying on extrapolations from Oman or Canada (20). This reliance on proxy data may not accurately reflect local epidemiology, particularly given the region’s unique demographic and healthcare structures. More localized surveillance is urgently needed. Across the region, multidrug-resistant *S. pneumoniae* strains are increasingly common, particularly in Kuwait and the UAE. In the UAE, the percentage of multidrug-resistant isolates nearly tripled from 2013 to 2021 (17, 18, 40, 41).

Mass gatherings such as the Hajj pilgrimage further complicate disease control. These events bring together diverse populations in densely crowded settings, increasing the risk of pneumococcal transmission. However, serotype-specific data during such gatherings are limited. Enhanced surveillance and targeted vaccination of high-risk attendees could mitigate this risk (35). Another pressing issue is the impact of expatriate populations on disease dynamics. The Gulf’s large, mobile expatriate workforce, often with varying vaccination statuses and healthcare access adds complexity to IPD epidemiology. Studies from Dubai and Abu Dhabi underscore higher disease burdens among expatriates and call for vaccination strategies inclusive of this population (17, 18, 20). Finally, diagnostic limitations may lead to underreporting or misclassification of IPD. Challenges in isolating *S. pneumoniae* from blood cultures, particularly in the UAE, have been noted and point to the need for investment in molecular diagnostic capabilities. In summary, while progress has been made in disease reduction through vaccination, the evolving epidemiology of IPD in the Gulf demands ongoing surveillance, broader vaccine adoption, enhanced AMR monitoring, and tailored public health strategies to address demographic and epidemiologic complexities unique to the region.

## Future directions

While this narrative review provides valuable insights into the epidemiology, burden, serotype distribution, and risk factors of IPD in the Gulf region, several gaps in knowledge may benefit from further research. Firstly, there is a paucity of recent data on IPD incidence and serotype distribution in some Gulf countries, highlighting the need for ongoing surveillance to monitor trends and inform vaccination strategies. Additionally, the impact of pneumococcal vaccination programs on disease burden and serotype epidemiology remains incompletely understood, necessitating long-term monitoring and evaluation studies. While vaccines like PCV13 have significantly reduced disease due to certain serotypes, there is a notable emergence of NVTs that may not be adequately covered by current formulations. For example, in Saudi Arabia, NVTs like 15 and 23A have been linked with invasive disease (57) and 24F has emerged in Kuwait (21), suggesting that broader vaccine coverage is needed. Ongoing surveillance of these NVTs and their epidemiological patterns will be critical in informing vaccine inclusion strategies and enhancing regional protection. Moreover, the emergence of AMR among pneumococcal strains underscores the importance of surveillance efforts to guide antibiotic stewardship programs and ensure effective treatment. Future research should also explore the impact of demographic, socioeconomic, and environmental factors on IPD risk, particularly in vulnerable populations such as children and the older adults as well as among the expatriate population. Lately, the Global Pneumococcal Sequencing (GPS) project, which involves whole genome sequencing of large number of isolates, especially those from low-and middle-income countries offers new directions for pneumococcal research. Some of the isolates from GCC countries are part of this project and it will be worthwhile to establish molecular typing of pneumococcal isolates from IPDs to understand the dynamics of IPDs and use the outcome for preventive aspect and vaccine serotype selection (7, 12, 17, 18, 21, 31, 33, 34, 42, 47). Lastly, comparative studies assessing the cost-effectiveness of different vaccination strategies, especially with the introduction of higher-valency vaccines such as PCV20 and the consideration of NVTs, will be essential to optimize IPD prevention and control efforts. Studies should also focus on the implementation of targeted interventions tailored to the Gulf region’s unique epidemiological context, ensuring that vaccination programs address the evolving burden of disease. Furthermore, future research should aim to establish clear links between vaccination programs, public health interventions, and observed epidemiological trends in the region.

## Limitations

Despite efforts to provide a comprehensive overview of IPD in the Gulf region, several limitations should be acknowledged. Firstly, the reliance on published literature may introduce selection bias, as studies with significant findings are more likely to be published, potentially overlooking unpublished data. Secondly, the inclusion criteria for studies may have inadvertently excluded relevant research conducted in the Gulf region, particularly if it was not indexed in the selected databases or published in English. Additionally, focusing solely on IPD may have excluded important insights into non-invasive pneumococcal disease, which also contributes to the overall burden. The heterogeneity of study designs, populations, and methodologies across the included studies may also limit the comparability and generalizability of findings. Furthermore, the focus on IPD may have overlooked important insights into non-invasive pneumococcal diseases, contributing to the overall burden of pneumococcal infections. Finally, the narrative synthesis approach employed in this review may lack the rigor of systematic reviews or meta-analyses, potentially impacting the robustness of conclusions drawn from the synthesized evidence.

## Conclusion

IPD poses a significant public health challenge in the Gulf region, with young children, the older adults, and individuals with chronic medical conditions being particularly vulnerable. Epidemiological data from the Gulf region underscores the significant burden of IPD, particularly among young children and high-risk populations. Despite the introduction of PCVs in several countries, challenges such as AMR and the emergence of NVTs persist.

Serotype distribution varies widely across the Gulf region, with certain serotypes like 19F, 23F, and 6B being prevalent. These trends suggest that higher-valency vaccines, such as PCV20, may help address the rise in NVTs and resistant strains. The emergence of non-PCV13 serotypes and the increasing prevalence of AMR further emphasize the need for continued surveillance and updated vaccination strategies tailored to the regional epidemiology. The findings highlight the need for enhanced surveillance to monitor serotype shifts and guide vaccine policy, particularly with newer vaccines offering broader coverage. Addressing AMR through antibiotic stewardship programs is crucial, given the rising rates of resistance observed in multiple studies across the region. Additionally, demographic factors such as the large expatriate population and socio-economic disparities further complicate disease control efforts. Moving forward, continuous monitoring of vaccine impact, targeted vaccination strategies, and a stronger emphasis from molecular epidemiology to genomic surveillance are essential to mitigate the burden of IPD. Expanding local research efforts and addressing gaps in knowledge about disease dynamics, particularly among vulnerable populations, will be key to optimizing prevention and treatment strategies in the Gulf region.
