# Supplementary material for: Invasive pneumococcal disease in the Gulf region: a narrative review of incidence, burden, and vaccine strategies
Source: Front Public Health. 2025 Sep 5;13:1589366. doi: 10.3389/fpubh.2025.1589366 (PMC12446325; doi:10.3389/fpubh.2025.1589366)
Supplement: Supplementary file 1 [file Table_1.docx]

Supplementary Table 1. Top non-invasive *Streptococcus pneumoniae* serotypes identified in different countries

| **Serotype** | **Prevalence/Characteristic** |
| --- | --- |
| 6C (Croney et al., 2013) | Found in ~4.8% of non-invasive isolates (large study of 1,662 serogroup 6 non-invasive samples) |
| 11A (Cleary et al., 2022) | Approximately 9.6% carriage in children, low invasiveness |
| 21 (Jacobs et al., 2009) | Common in non-invasive isolates, including respiratory tract infections |
| 17F (Olarte et al., 2017) | Included in PCV21, important for non-invasive pneumococcal pneumonia and AOM |
| 23A (Mendes et al., 2015) | Common non-invasive serotype in respiratory infections |
